# Photo-Induced Drug Release from Polymeric Micelles and Liposomes: Phototriggering Mechanisms in Drug Delivery Systems

**DOI:** 10.3390/polym14071286

**Published:** 2022-03-23

**Authors:** Najla M. Salkho, Nahid S. Awad, William G. Pitt, Ghaleb A. Husseini

**Affiliations:** 1 Department of Chemical Engineering, College of Engineering, American University of Sharjah, Sharjah P.O. Box 26666, United Arab Emirates; nsalkho@aus.edu (N.M.S.); nawad@aus.edu (N.S.A.); 2 Materials Science and Engineering Program, College of Arts and Sciences, American University of Sharjah, Sharjah P.O. Box. 26666, United Arab Emirates; 3 Chemical Engineering Department, Brigham Young University, Provo, UT 84602, USA; pitt@byu.edu

**Keywords:** chemotherapy, photoresponsive, light, nanocarriers, triggering release

## Abstract

Chemotherapeutic drugs are highly effective in treating cancer. However, the side effects associated with this treatment lower the quality of life of cancer patients. Smart nanocarriers are able to encapsulate these drugs to deliver them to tumors while reducing their contact with the healthy cells and the subsequent side effects. Upon reaching their target, the release of the encapsulated drugs should be carefully controlled to achieve therapeutic levels at the required time. Light is one of the promising triggering mechanisms used as external stimuli to trigger drug release from the light-responsive nanocarriers. Photo-induced drug release can be achieved at a wide range of wavelengths: UV, visible, and NIR depending on many factors. In this review, photo-induced release mechanisms were summarized, focusing on liposomes and micelles. In general, light-triggering mechanisms are based on one of the following: changing the hydrophobicity of a nanocarrier constituent(s) to make it more soluble, introducing local defects within a nanocarrier (by conformational transformation or photo-cleavage of its lipids/polymers chains) to make it more porous or concentrating heat for thermo-sensitive nanocarriers to release their payload. Several research studies were also presented to explore the potentials and limitations of this promising drug release triggering mechanism.

## 1. Introduction

Although nanosized carriers have been used effectively in drug delivery, ongoing studies aim to improve their targeting efficiency and drug release kinetics. Nanocarriers used in drug delivery received particular attention in cancer treatment as part of the efforts seeking to overcome the systemic toxicity resulting from the lack of specificity associated with conventional chemotherapy. In general, nanocarriers are classified into two categories: organic and inorganic. Organic nanocarriers include micelles, liposomes, solid-lipid nanoparticles, dendrimers, and nanoemulsions; inorganic nanocarriers include gold nanoparticles, superparamagnetic iron oxide nanoparticles (SPIONS), metal organic frameworks, carbon nanotubes, silica nanoparticles, and quantum dots. These delivery vehicles are designed mainly to reduce the systemic side-effects of drugs by retention through encapsulation, thus sparing non-targeted body tissues from high drug exposure and maintaining therapeutic levels by depositing the loaded drug only at the diseased site. Among all the nanocarriers, liposomes are the most attractive carrier in drug delivery systems due to their self-assembly, stability, and their ability to encapsulate hydrophilic and hydrophobic drugs [1]. There are several methods well documented in literature to prepare liposomes, such as thin-film hydration, sonication, ethanol injection, reverse-phase evaporation, and others. These methods are used in small-scale production, and they usually require organic solvents/surfactants during synthesis. The undesirable residue from organic solvents/surfactants in liposome formulations and the heterogeneous size distribution are considered the major drawbacks of these conventional methods [2]. Supercritical CO_2_ (SC-CO_2_) is an innovative technique that produces liposomes at a large scale without using organic solvents. The technique improves the encapsulation efficiency and results in a uniform size distribution without sonication or extrusion. Briefly, CO_2_ is mixed with the lipidic solution, where they enter a chamber with atomized water droplets. Due to the high diffusion of CO_2_ and the reduced viscosity of the lipidic solution, lipids will coat water droplets at a fast rate resulting in an inverted micelle-like structures [3]. Inverted micelles are further stabilized by another layer of lipids once they fall into a bulk of water placed at the bottom of the chamber [3]. The technique improves the reproducibility of liposomes with a uniform size distribution. Chaves et al. [3] used the supercritical-assisted liposomes (SuperLip) method to encapsulate vitamin D_3_ with high encapsulation efficiency. For more studies on this technique refer to [2,3,4,5].

Tumor tissues have unique features that can help deliver drug loaded-nanocarriers to these targeted sites. One of these useful features is the deformed tumor vasculature known as the enhanced permeability and retention (EPR) effect. Nanocarriers with small sizes (~100–400 nm) [6] can extravasate through the leaky tumor vasculature and accumulate inside the tumor tissues due to poor lymphatic drainage. This is known as the passive targeting of tumors. Although the EPR effect can be exploited for the accumulation of nanodrugs in the tumor, this effect alone is not sufficient; usually, the EPR effect achieves around less than a 2-fold increase of drug delivery to the tumor compared to normal tissues [7]. Hence, biomarkers can be conjugated to the nanocarrier’s surface to match specific receptors overexpressed on cancer cells to improve the targetability. This is known as the active targeting of tumors. Those smart nanocarriers can be designed to be responsive to internal or external stimuli to trigger the release of the encapsulated drug in a controlled manner upon their accumulation inside the targeted tumor. Nanocarriers responsive to internal stimuli benefit from different tumor characteristics such as the acidic pH, higher temperature as well as enzymatic and redox activities. However, internal stimuli are generally slow and difficult to control [8]. On the other hand, external triggers such as ultrasound, magnetic field, and light are more tunable for a sustained drug release both spatially and temporally. Table 1 compares the advantages and challenges of the aforementioned triggering techniques.

**Figure 1:**
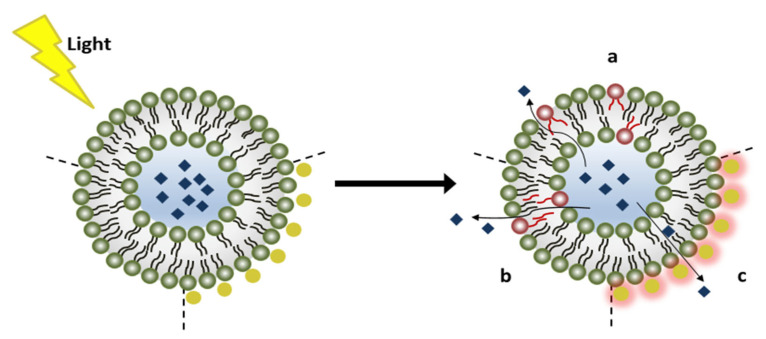
Photo-induced mechanisms used in triggering drug release from nanocarriers. (a) Photo-isomerization; (b) Photo-cleavage; (c) SPR of Gold NPs for thermo-sensitive nanocarriers, gold NPs can be on the surface or inside.

In this review, light-triggering mechanisms in drug delivery systems will be presented. Light is a relatively safe electromagnetic radiation that can be used to trigger drug release from nanocarriers, including micelles, liposomes, metal nanoparticles, and nanogels. Furthermore, light can be focused on a specific location in the body, allowing for accurate spatiotemporal drug release from the nanocarriers. Various light parameters can be adjusted to achieve the desired effect of drug release from the nanocarriers; these parameters include the wavelength, intensity, exposure duration, and beam diameter (focus) [22]. Light penetration in tissues depends on the wavelength; radiation with wavelengths below 650 nm can penetrate a maximum of 1 cm deep because of high scattering and absorption by hemoglobin, oxy-hemoglobin, and water [22]. Radiation with wavelengths near-infrared (NIR) in the range of 650–900 nm can penetrate up to 10 cm deep [22]. Light has been used to release the payload of nanocarriers using UV, visible, and NIR radiation. The latter is preferable in drug delivery because they are safer on tissues and can penetrate deeper. The different photo-induced mechanisms that can be exploited to release encapsulated payloads from nanocarriers are photo-isomerization, photo-cleavage, surface plasmon resonance (SPR) absorption, hydrophobicity change, and de-crosslinking. Some of these mechanisms are shown in Figure 1.

## 2. Mechanisms of Photo-Induced Drug Release from Nanocarriers

### 2.1. Photo-Isomerization

**Figure 2:**
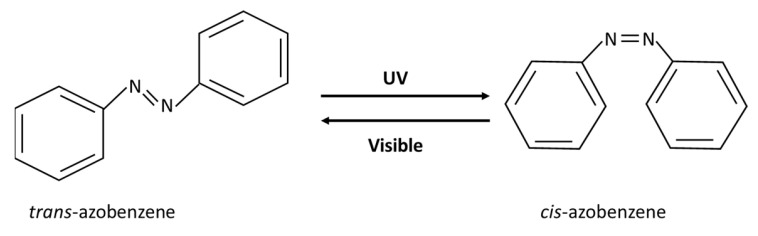
Azobenzene photo-isomerization.

Photo-induced isomerization is achieved by a conformational change around a restricted bond such as a carbon double bond. The goal is to change the conformation from *trans* to *cis*. Isomers with *trans* orientation are more tightly packed than those with *cis* orientation. Therefore, upon exposure to UV light, a conformational change from the *trans* to *cis* form may make a nanocarrier more permeable and may disrupt its structural integrity. This is due to both the steric effect and the increased polarity of the *cis* form [23]. This mechanism has been used to release the payload of a nanocarrier. Azobenzene shows this type of photoresponsive behavior and is thus referred to as a “photoswitchable chemical”. Azobenzene has two phenyl rings linked with a double bond between nitrogen atoms, as shown in Figure 2. When irradiated with UV light at wavelengths between 320–350 nm, azobenzene undergoes a reversible isomerization from *trans* to *cis* at the double bond between the nitrogen atoms. Subsequently, when exposed to visible light (400–450 nm) or heat, azobenzene reverts to the *trans* form. In addition to azobenzene, other photoresponsive moieties have been used in drug delivery systems (DDSs), such as retinoyl, spiropyran and stilbene [23].

A study conducted by Liu et al. [24] examined the effect of different polarities of three cholesterol-azobenzene lipidic derivatives when irradiated with UV light. Among the three lipidic derivatives, AB lipid 3 showed the highest photo-isomerization in in-vitro studies. It was found that 90% of AB lipid 3 suspended in chloroform converted from *trans* to *cis* when irradiated with UV light at 360 nm for 6 min. On the other hand, when AB lipid 3 was impeded in liposomes based on phosphatidyl choline (PC), it was found that only 65% converted from *trans*-AB lipid 3 to *cis*-AB lipid 3 when irradiated with UV light for 10 min [24]. The reduction in photo-isomerization of AB lipid 3 in liposomes indicated that it was successfully incorporated into liposomes. The mechanism of *trans* to *cis* transformation of AB lipid 3 was exploited in in-vitro release experiments on PC-based liposomes encapsulating calcein as a model drug. In one of the release experiments, liposomes incorporating AB lipid 3 were suspended in PBS buffer, and then the sample was irradiated periodically every 4 h with UV light (10 min) and visible light (15 min) at 15 °C. The release of calcein was measured by fluorescence spectroscopy. At an optimized molar ratio of 4:1 (PC: AB lipid 3), the release from the photo-triggered liposomes was double the spontaneous release after 40 h when liposomes were irradiated periodically with UV-visible light [24]. UV exposure successfully showed increased calcein release, while visible light suppressed the release as the lipids reverted from the *cis* form to the *trans* form.

**Figure 3:**
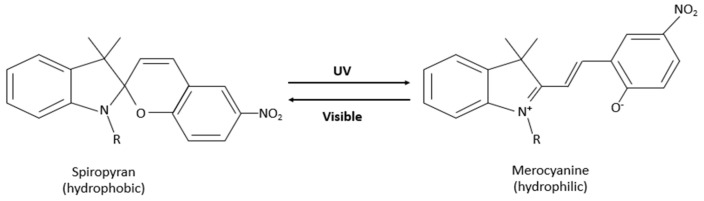
Photo-isomerization of Spiropyran and Merocyanine.

Spiropyran (SP) is another light-responsive molecule. It is nonpolar, hydrophobic, and colorless. When irradiated with UV light (200–400 nm), closed-neutral SP converts, by ring-opening through a *cis-trans* isomerization, to zwitterionic merocyanine (MC), which is hydrophilic and colorful. The isomerization can be reversed thermally or by visible light irradiation (500–600 nm), as shown in Figure 3 [25,26]. The MC molecule has high hydrophilicity; it dissolves in aqueous solutions. The SP-MC isomerization was utilized in drug delivery, especially in polymeric micelles, along with other applications. Research conducted by Shen et al. [25] studied the light-triggered release of Dox from SP-PMPC polymeric micelles. The Dox release was assessed in-vitro using the dialysis bag method and the analysis of the UV-Vis spectra of Dox (λ = 485 nm) from the samples withdrawn at predetermined time intervals. When SP-PMPC micelles were irradiated with UV light at λ = 365 nm for 20 min, researchers reported Dox release of 50%, which is significantly higher than the release from control liposomes (Dox release of 20%) after 24 h [25]. The change in the structure of micelles that is the reversible self-assembly and disassembly was the primary mechanism behind drug release after exposure to UV/Vis light. Furthermore, Shen et al. [25] reported that the diameter of micelles reduced from 37.2 to 4.7 nm after UV irradiation for 10 min. This was explained by the disassembly of SP-PMPC micelles, especially since the MC-PMPC conjugate is more hydrophilic. And when MC-PMPC was irradiated with visible light, the diameter increased from 4.7 to 40.6 nm, thus supporting the reversible formation of micelles [25]. Those findings were further confirmed by transmission electron microscopy (TEM). 

### 2.2. Photo-Cleavage

**Figure 4:**
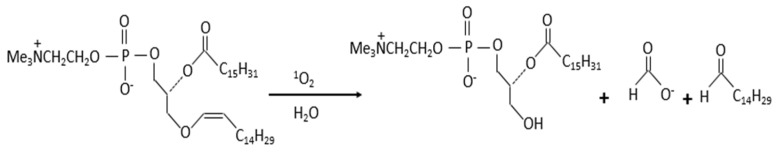
Photo-oxidation of plasmalogen at the vinyl ether linkage mediated by singlet oxygen.

Photo-cleavage through photosensitization-induced oxidation in nanocarriers can be achieved when a photosensitizer (PS) and oxygen are in proximity to an oxidizable lipid. The nanocarrier used in this mechanism should have a lipid segment sensitive to singlet oxygen produced by a PS within a suitable distance [27]. Using this mechanism, drug release from the nanocarrier occurs by creating local defects that lead to membrane destabilization [28]. When irradiated with light, photosensitizing agents absorb photons and are excited to a triplet state; this generates reactive oxygen species (ROS) that are radicals (such as hydroxyl and superoxide) or nonradicals (such as singlet oxygen) [27]. An excited PS is usually used in drug delivery to produce a highly reactive singlet oxygen ^1^O_2_ by transferring energy to a ground-state molecular oxygen ^3^O_2_ [28]. The singlet oxygen is an excited state of oxygen where the spin of one of the unpaired electrons is inverted to yield electrons spinning in the opposite direction. Singlet oxygen has a concise life of 10^−5^ s, and it is a strong oxidant that can oxidize cellular constituents such as proteins, lipids, and nucleic acids [29]. The diffusion distance of ^1^O_2_ is short but has sufficient energy to react at any site within a 100 nm liposome, as reported by Gerasimov et al. [30]. Anderson and Thompson first reported drug release from liposomes using photo-induced oxidation [31] in 1992. In this study, glucose was released from liposomes made of semi-synthetic plasmalogen, a class of glycerophospholipids, which was triggered using visible light (λ > 640 nm) and activated with zinc phthalocyanine (ZnPc) as a photosensitizer. ZnPc was inserted within the hydrophobic interior of the liposomal membrane. Anderson and Thompson [31] reported a release of 62% of encapsulated glucose at 37 °C in 60 min, double the release from control liposomes under dark conditions. By irradiating ZnPc in an air-saturated medium, singlet oxygen is formed, which oxidizes plasmalogen at the vinyl ether linkage, as shown in Figure 4 [22,32]. The cleavage of the vinyl ether linkage results in the formation of single-chain lipid, which introduces local defects in the membrane, hence increasing its permeability to glucose [22].

In addition to the vinyl ether linkage, other structures are reactive with ^1^O_2_. For example, in DDSs activated by NIR light, some of the sensitive structures reported in the literature are olefin linkers (lipids, vinyl disulfide, vinyl ether, and aminoacrylate), thioketal linkers, polymers containing selenium, tellurium, or poly(propylene sulfide), and imidazole derivatives [27]. Refer to Dariva et al. [27] for further details about the reaction of ^1^O_2_ with these types of linkers.

**Figure 5:**
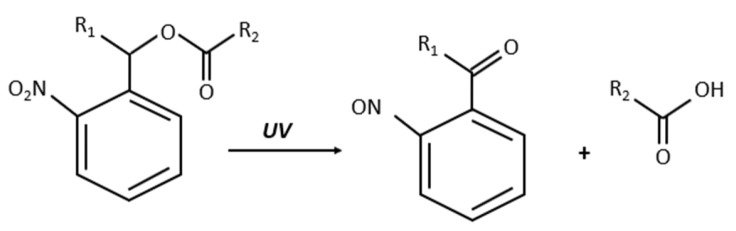
Photo-cleavable *o*-nitrobenzyl ester irradiated with UV light.

Coumarin and *o*-nitrobenzyl groups are common photo-cleavable linkers used in drug delivery systems. These linkers decompose upon irradiation with either UV or NIR light. For example, Figure 5 shows the photo-cleavable reaction of *o*-nitrobenzyl ester when irradiated with UV light [33].

A study conducted by Zhang et al. [34] investigated the photo-cleavage of the *o*-nitrobenzyl derivative in liposomes. They synthesized liposomes from triazole and *o*-nitrobenzyl-containing phosphatidylcholine (TNBPC) lipids. When irradiated with UV light, the *o*-nitrobenzyl cleaves one hydrophobic chain, which introduces defects that increase the permeability of the bilayer membrane of liposomes. The study assessed the permeability of TNBPC liposomes in comparison to control liposomes (TBPC) without the photolabile *o*-nitrobenzyl group. The permeability study was performed in-vitro by loading liposomes with 8-hydroxy-1,3,6-pyrenetrisulfonate (HPTS). HPTS is a pH-sensitive fluorescent probe. Loaded liposomes were suspended in HEPES buffer in a fluorescence cuvette and the sample was irradiated with UV light. Irradiating both groups of liposomes with UV light (λ = 320–390 nm) at 10 mW/cm^2^ for 80 min resulted in a 70% increase in the permeability of TNBPC liposomes after 60 min, which is twice the permeability of TBPC liposomes. Interestingly, while photo-cleavage was behind the increase in permeability of the TNBPC liposomes, the control TBPC liposomes responded to UV light by a different mechanism––mainly a fusion of TBPC liposomes caused the partial release of HPTS when irradiated with light, as confirmed by microscopy studies. During the fusion of liposomes, some HPTS leaked, but the overall liposome structure remained intact in the control liposomes.

Photo-cleavage was also used in studies with polymeric micelles. Research conducted by Yan et al. [35] used NIR light to cleave the hydrophobic core of micelles and release the encapsulated model drug Nile Red (NR). The micelles were made of di-block copolymer poly(ethylene oxide) (PEO) as the hydrophilic part and polymethacrylate bearing photolabile *o*-nitrobenzyl group (PNBMA) as the hydrophobic part. To enable the cleavage of micelles through the *o*-nitrobenzyl group, Yan et al., utilized upconverting nanoparticles (UCNPs) made of NaYF4:TmYb to convert NIR to UV light, which in turn cleaves the *o*-nitrobenzyl group. UCNPs are optical materials doped with lanthanide ions that undergo electronic transitions within the 4f electron shells. They have been used in many applications such as deep-tissue bioimaging, fluorescent microscopy, and more [36]. UCNPs are unique since they can convert two or more low-energy photons into one high-energy photon; thus, they can convert photons absorbed in the NIR to photons emitted in the visible or UV regions. Micelles prepared by Yan et al. [35] were loaded with UCNPs and NR, the latter being the model drug. While the study was not aimed to optimize the conditions of release, researchers proved that irradiating micelles with NIR light was able to disrupt them and that the release was dependent on the amount of UNCPs and the intensity of the NIR light. Two experiments were performed in-vitro using the dialysis membrane method to investigate the disruption of micelles. First, UCNP-loaded micelles (without NR) were irradiated with NIR light, and the absorption spectra change over time was detected for the photocleaved nitrosobenzaldehyde molecules. Over time, the increase in absorbance confirmed the photocleavage of *o*-nitrobenzyl moieties by NIR light. Second, micelles loaded with both UCNPs and NR were irradiated with NIR, and the decrease in fluorescence of NR was detected over time. The quenching of NR fluorescence indicated its release from micelles. The results established that UCNPs converted two or more NIR photons to one higher energy photon in the UV region that was absorbed by *o*-nitrobenzyl group; the cleavage of the latter was able to dissociate micelles and release the encapsulated model drug. Micelles disruption was further validated by TEM in their paper [35].

### 2.3. Surface Plasmon Resonance Absorption

**Figure 6:**
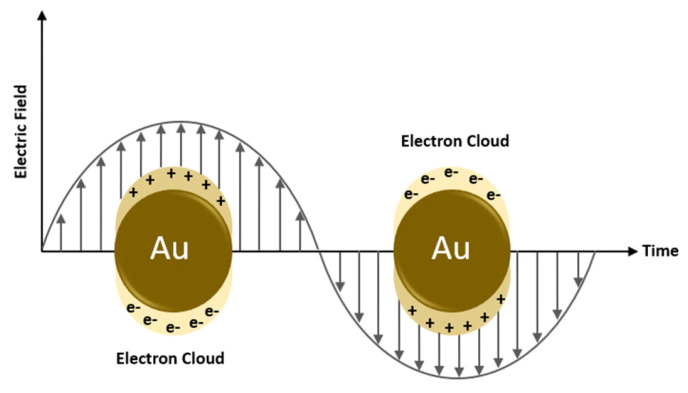
Surface plasmon resonance.

**Figure 7:**
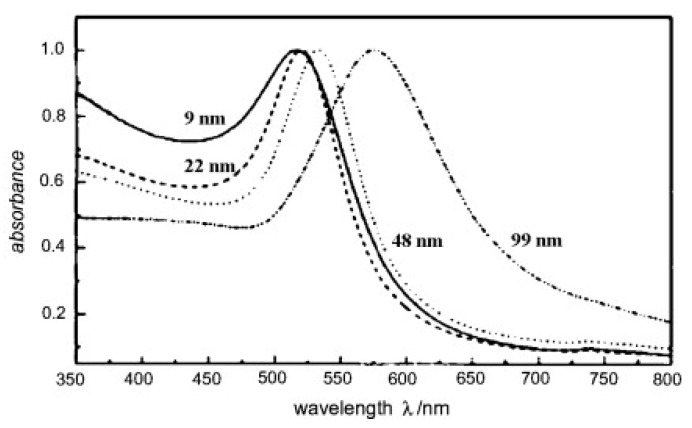
Surface plasmon resonance for different sizes of gold nanoparticles. Reprinted with permission from [38]. Copyright 1999 American Chemical Society.

Some metals exhibit unique optical properties when present in the nanoparticle form, compared to their bulk properties. Gold is one of these metals exhibiting different absorption frequencies depending on the nanoparticle size and shape. When a metallic nanoparticle such as gold is exposed to light, free electrons oscillate in response to the oscillating electromagnetic field of the incident light. The coherent oscillation of electrons on the metal surface causes a slight separation of net charges, which induces a dipole along the direction of the electric field of the light, as shown in Figure 6 [37]. At a specific frequency/wavelength, termed the surface plasmon resonance (SPR), the amplitude of the oscillation reaches a maximum which correlates to a high absorbance of incident light as measured by a UV-Vis spectrometer [37]. The SPR frequency and intensity for a specific metal depend on the electron charge density that is affected by many factors such as size, shape, composition, structure, and the dielectric constant of the surrounding medium [37]. The effect of the size of gold nanoparticles on the SPR wavelength is depicted in Figure 7. 

When electromagnetic waves illuminate such a nanoparticle, part of its energy is lost due to light absorption and/or scattering. Light is absorbed when the energy of a photon is dissipated in an inelastic process [37]. On the other hand, light is scattered when the energy of a photon causes electrons to oscillate, which in turn emit photons in the form of scattered light either at the same frequency of the incident light or at a shifted frequency [37]. The Mie theory explains surface plasmon’s absorbance, scattering, and total extinction efficiencies. A study conducted by Huang and El-Sayed [37] investigated the optical absorption and scattering of gold nanoparticles as a function of their size based on full Mie theory. At a small nanoparticle size of 20 nm, the authors found that the extinction is due to absorption only. Whereas with increasing sizes of 40 and 80 nm, authors reported that the extinction results from absorption and scattering contributions. Those findings can help select the appropriate size of gold nanoparticles for the application. For example, imaging applications require high scattering efficiency; thus, nanoparticles of larger sizes are suitable. On the other hand, in applications that require a photothermal effect such as cell destruction, nanoparticles should absorb light efficiently and convert it to heat; hence, smaller nanoparticles are more desirable for thermal activation, such as in drug delivery.

Gold nanoparticles have been used in cancer therapy, and some recent research explores their use in drug release from thermo-sensitive nanocarriers. Basically, photon energy is converted to thermal energy by the SPR effect in metals nanoparticles. When a metal nanocarrier is excited by light at a specific wavelength, an energetic surface plasmon is formed (Figure 6) that very rapidly cools down (~1 ps) by transferring heat to the nanoparticle lattice [22]. The nanoparticle lattice exchanges this heat with the surroundings within ~100 ps, thus causing localized heating [22]. This localized heating effect can be used to impose thermal and mechanical stresses on nanocarriers to release their payload. Figure 7 shows that gold nanoparticles can absorb light efficiently by the SPR effect in the visible region at various wavelengths depending on their size. In addition to the size of a nanoparticle, other factors such as shape (rod v sphere, hollow v solid) can also affect the wavelength at which a gold nanoparticle absorbs light, as mentioned earlier. This tunable optical resonance of gold can be used to prepare nanoparticles with high absorption in the NIR region by tailoring their properties, including size, shape, and structure. 

Recent research conducted by Moretti et al. [39] investigated drug release from a compound hydrogel made of agarose and carbomer 974P and loaded with gold nanoparticles. By utilizing the surface plasmon resonance effect of the gold nanoparticles, those nanoparticles act as nuclei to collect and convert light to heat, enabling thermally-induced drug release from the hydrogel matrix. The mechanism of drug release is based on the expansion of the mesh size of the hydrogel matrix due to localized heating, thus creating free space in the gel matrix and allowing the entrapped drug molecules to diffuse from the hydrogel [39]. The increased release due to the SPR effect was noticeable when a model drug (fluorescein dextran (DEX)) had a larger size than the original mesh size of the hydrogel before irradiation with light. Moretti et al. [39] also studied the effect of surface modification of the gold nanoparticles by attaching PEG molecules to stabilize the particles, thus preventing aggregation, referred to as “isolated particles” in the paper. The release profiles of DEX from PEGylated (isolated) and non-PEGylated (aggregated) gold NPs in hydrogels differed. Researchers found that hydrogels with aggregated gold NPs showed higher and faster release kinetics of ~100% within one hour of irradiation. In contrast, hydrogels with isolated gold NPs reached only 25% drug release after the first hour of irradiation. This was explained by the more significant localized heating effect when the gold NPs were not isolated [39].

A study conducted by Montoto et al. [40] examined drug release from gold nanostars (AuNSts) coated with a mesoporous silica shell, capped with paraffin that acts as a thermosensitive molecular gate and loaded with doxorubicin (AuNSt@mSiO_2_@Dox@paraffin). A 96-well plate was used to fill the nanoparticle suspensions. Upon irradiation with NIR light (λ = 808 nm) that matches the SPR of the gold nanoparticles and at a power density of 4 W/cm^2^, nearly 30% of the drug was released within 20 min, according to the fluorescence spectra of Dox. In contrast, the non-irradiated nanoparticles showed negligible release [40]. Furthermore, the temperature of the irradiated sample reached 49 °C after 15 min. The temperature increase above the melting point of paraffin resulted in uncapping the mesoporous silica, which acts as gates to drug diffusion and release. In addition, cells viability studies were conducted using HeLa cells for cervical cancer, and they showed promising results.

### 2.4. Hydrophobicity Change

Polymeric micelles are made of amphiphile block copolymers that maintain their stability when their concentration is above a specific concentration called the critical micelle concentration (CMC). If the concentration of polymers falls below the CMC value, micelles tend to disintegrate, thus releasing their content. Unfortunately, this mechanism is difficult to employ in drug release from micelles. When micelles enter the bloodstream by IV injection, they are diluted below their CMC and may not retain their payload by the time they reach the desired site. Often this is considered a thermodynamic instability issue. If the CMC could be decreased, the payload could be delivered after the micelles reach the target site. Several techniques were developed to decrease the CMC value of micelles to improve their thermodynamic stability. Although higher stability of micelles preserves the encapsulated drug, a release mechanism should be designed to eliminate this stability at the target site by raising the CMC.

To modulate the CMC, a photochemical reaction method was developed to change the hydrophobicity of molecules, increasing the CMC and releasing the payload from micelles in a controlled manner. The release mechanism is based on converting the amphiphilic polymers to a more hydrophilic form that increases the CMC and dissolves the micelles, thus providing hydrophobic drug release. Light-sensitive molecules such as organic chromophores can be incorporated into micelles where the NIR-induced chemical transformation to a hydrophilic form can be used to shift the CMC of micelles and thus control the release of their hydrophobic payloads. Most of the organic chromophores are utilized to absorb UV light to generate charged species (more hydrophilic) through a photochemical transformation. However, NIR light (λ = 650–900 nm) is more convenient for medical applications since light can penetrate deeper into tissues, up to 10 cm, with a lower risk of tissue damage [22]. A few organic chromophores were found to achieve the same chemical transformation induced by one-photon UV-light absorption when irradiated with NIR light but through two-photon absorption [22].

**Figure 8:**
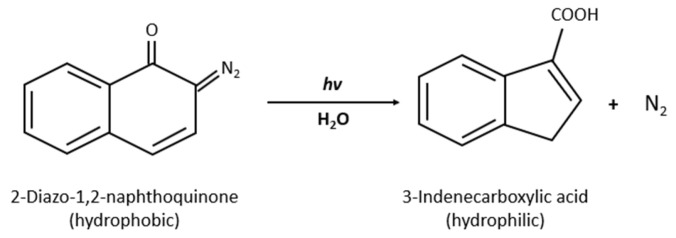
Hydrophobic to hydrophilic change of DNQ after Wolff rearrangement induced by UV/NIR light in buffered water.

Goodwin et al. [41] synthesized micelles from amphiphilic molecules made of hydrophilic PEG conjugated to a hydrophobic 2-Diazo-1,2-naphthoquinone (DNQ). DNQ is light sensitive, and when irradiated with UV or NIR light, it undergoes a Wolff rearrangement that forms a hydrophilic derivative, namely, the 3-indenecarboxylic acid (Figure 8), which dissolves micelles in the aqueous solution, thus releasing their content. Goodwin et al., studied the release of Nile Red (NR) in-vitro from PEG-DNQ micelles above and below the CMC upon irradiation with UV light at 350 nm. The UV absorbance and fluorescence emission spectra were measured at different time intervals. Above and below the CMC, the UV absorption at two peak wavelengths showed a decrease with increasing irradiation time, indicating the conversion of DNQ to 3-indenecarboxylate. And while the fluorescence emission spectra of NR decreased with increasing irradiation time for the sample prepared above the CMC, it barely changed for the sample prepared below the CMC, and instead, it remained at low fluorescence. The authors concluded that the release of NR was due to the destruction of micelles triggered by the photoreaction of UV light. In addition, the disassembly of micelles was confirmed by the quenching of Nile Red fluorescence that was encapsulated in micelles when irradiated with NIR light at 795 nm [41].

A study published recently has expanded the work of Goodwin et al. [41] using a similar micellar formulation with DNQ as a ligand [42]. In this study, micelles were loaded with docetaxel (DTX) as an anti-cancer drug to assess the cytotoxicity of the micellar formulation when irradiated with UV light. Authors incubated MCF-7 (breast cancer cell line) cells with targeted micelles (NPDNQ) both empty and loaded with docetaxel: NPDNQ and DTX-NPDNQ. When irradiated with UV light at λ = 395 nm (50 mW/cm^2^) for 10 min and after an incubation of 24 h, cells viability of DTX-NPDNQ decreased from 55% (before irradiation) to 36% (after irradiation) [42]. On the other hand, empty NPDNQ showed no significant difference in cells viability before and after irradiation. The authors concluded that the disruption of micelles achieved cytotoxicity through the Wolff rearrangement that converted the hydrophobic core to a hydrophilic form, thus solubilizing the micelles. This was further confirmed by a study of micelles’ hydrodynamic size, which reflected a significant decrease in micelles size after being irradiated with UV light [42].

### 2.5. De-Crosslinking

**Figure 9:**
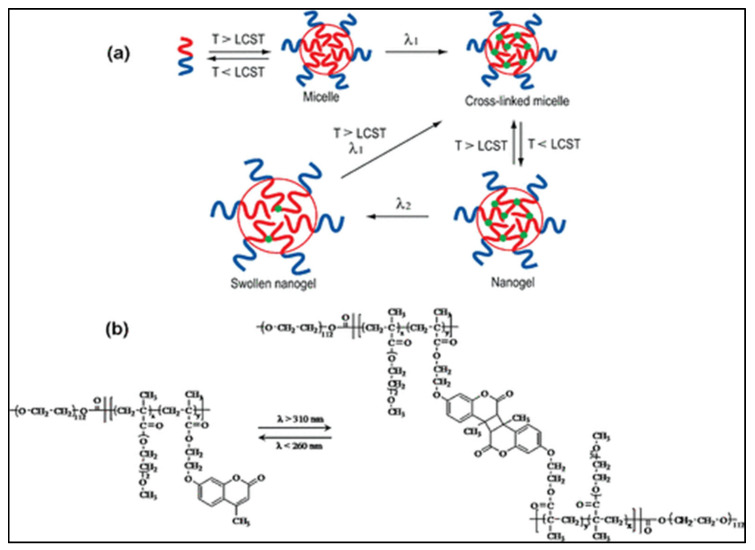
(**a**) Preparation of nanogel particles through micelle formation at T > LCST, followed by crosslinking of coumarin by UV irradiation at λ_1_ > 310 nm. De-crosslinking was achieved by irradiation at λ_2_ < 260 nm; (**b**) Reversible photo-crosslinking of di-block copolymer PEO-*b*-P(MEOMA-*co*-CMA). Reprinted with permission from [43]. Copyright 2009 American Chemical Society.

Some nanoparticles, such as micelles, require crosslinking to improve their stability and retain their payload. Designing nanocarriers with photo-sensitive crosslinks enable their usage in the spatiotemporal release of drugs. Many studies used photo-crosslinking to form persistent micelles and nanogels. However, very few reported the usage of photo de-crosslinking in drug delivery. Reversible photo-de-crosslinking of nanogels was first reported in 2009 by He et al. [43] who synthesized nanogels from di-block copolymers made of poly(ethylene oxide) (PEO) and poly[2 -(2-methoxyethoxy) ethyl methacrylate-*co*-4-methyl-[7-(methacryloyl)oxyethyloxy] coumarin] (PEO-*b*-P(MEOMA-*co*-CMA)). Crosslinking was achieved by UV light (λ > 310 nm) through dimerization of coumarin above the lower critical solution temperature (LCST) of the P(MEOMA-*co*-CMA) polymer. To dissolve the nanogels, de-crosslinking of cyclobutane rings of coumarin was achieved by irradiating at λ < 260 nm (see Figure 9). De-crosslinking allows nanoparticles to swell, which can be reversed again by irradiating the swollen nanogels at λ > 310 nm, thus de-swelling the nanogel particles. The results of this study can be further explored in macro hydrogels to control the release of their payloads.

Lu et al. [44] synthesized microgels named [poly(MEO_2_MA-*co*-MAA-*co*-CMA)] composed of 2-(2-methoxyethoxy)ethyl methacrylate (MEO_2_MA), methacrylic acid (MAA) and 7-(2-mehacryloyloxyethoxy)-4-methylcoumarin (CMA). Those microgels were responsive to pH, temperature, and UV irradiation. And similar to He et al. [43], Lu et al., used reversible photo-crosslinking and photo-de-crosslinking by UV irradiation at λ = 365 nm and λ = 254 nm, respectively, to operate on the coumarin derivative segment of the polymer. This allowed a gradual and controlled release of encapsulated Dox.

### 2.6. Summary of Studies

In addition to the studies discussed previously under each mechanism, Table 2 below compiles additional studies that utilize phototriggering to release the payloads from micelles and liposomes.

## 3. Photodynamic Therapy and Nanomedicine in Cancer Treatment

Photodynamic therapy (PDT) is one of the treatment modalities of cancer. PDT is based on using light to activate photosensitizing (PS) agents, which generate radical oxygen species (ROS) or singlet oxygen (^1^O_2_), as explained in Section 2.2. Those species are highly reactive and can mediate damage to cells and tumors. PDT was first used in the late 1970s to treat patients with bladder cancer [56,57]. Later, many clinical studies were conducted to treat other types of cancer using PDT, and many PSs have been approved for clinical use; for more details, refer to [58]. As mentioned earlier, light can penetrate the skin by several millimeters and up to 1 cm only; therefore, PDT is limited to superficial tumors such as skin, lung, bladder, esophagus, and brain tumors. Among all, PDT is one of the best treatment modalities for skin cancer. Despite being non-invasive, PDT has a few limitations that restrict its application in cancer treatment, such as poor bioavailability, the unwanted side effect of some hydrophobic PSs, high dosage requirement, and self-aggregation in aqueous biological media due to the hydrophobic nature of most the PSs [59]. To improve the efficiency of PDT, nanocarriers can be used to encapsulate PSs and improve their bioavailability and stability. Limited in-vivo studies were reported for the combined PDT with nanomedicine in the field of cancer treatment, some of which are discussed below. For more studies, please refer to Table 3.

Visudyne^®^ (Novartis, Basel, Switzerland) is a liposomal formulation loaded with verteporfin; it was the first nanoformulation of a photosensitizer. Visudyne^®^ was approved by the FDA in 2000 to treat age-related macular degeneration (AMD) [60]. Verteporfin, the active ingredient in Visudyne, is photoactivated at λ = 692 nm [60]. Huggett et al. [61] conducted clinical trials (phase I/II) with verteporfin (non-liposomal formulation) to treat patients with advanced pancreatic cancer. Verteporfin was injected intravenously at a dose of 0.4 mg/kg body weight. Laser fibers (single and multiple) achieved the activation of the PS positioned percutaneously by a needle close to the tumor under computed tomography. After 60–90 min of verteporfin injection, several light doses were tested to achieve a target of 12 mm induced necrosis. The study successfully achieved 12-mm lesions in patients who received a light dose of 40 J/cm (single laser fiber), but it resulted in varying necrosis volume among patients. The results were promising in PDT-induced necrosis for pancreatic cancer. In addition, inserting laser fibers close to tumor overcame the problem of limited light penetration when applied outside the body.

Ichikawa et al. [62] examined the liposomal formulation Visudyne^®^ both in-vitro and in-vivo. In in-vivo studies, tumor-bearing BALB/c mice (Meth-A sarcoma) were injected with Visudyne^®^ intravenously at a dose of 0.25 mg/kg. The tumor site was then irradiated with laser light at λ = 689 nm (150 J/cm^2^, 0.25 W) 15 min or 3 h post-injection. Compared to a control group injected with saline, a higher suppression of tumor growth was observed in mice treated with Visudyne^®^ and irradiated with light. The highest tumor growth suppression was achieved when the light was activated after 15 min post-injection as opposed to 3 h post-injection. And by increasing the dosage of Visudyne^®^ to 0.5 mg/kg and irradiating with light after 15 min, it was reported that the tumor was completely suppressed in 60% of treated mice. The biodistribution of Visudyne^®^ was also assessed; for further details, please refer to [62].

Foslip^®^ (Sanofi-Aventis, Paris, France) is a liposomal formulation of a chlorine-derivative PS, namely m-THPC (temoporfin, trade name Foscan^®^). Foscan PS is used in the PDT treatment of squamous cell carcinoma of the neck and head; it was approved by the European Medsicines Agency in 2001 but was declined by the FDA. Foscan is photoactivated at λ = 652 nm, and it is more effective in generating singlet oxygen species than other PSs [63]. This formulation was used in many clinical trials to treat head, neck, and oral cavity tumors. Recently, Lambert et al. [64] published a comprehensive report for clinical trials using temoporfin-PDT to treat oral or oropharyngeal head and neck squamous cell carcinoma. The study included twenty-six patients who received their temoporfin-PDT treatment between 2002 and 2019 at the University Hospitals in Leuven, Belgium. In brief, the treatment protocol includes the intravenous administration of temoporfin, where the tumor was illuminated with laser (λ = 652 nm) 72–120 h post-injection. Patients were followed up clinically, and they were repeatedly examined for up to 10 years. The outcomes of the study reflected a complete tumor regression response in 76.9% of the treated patients. However, during a median follow-up period of 27 months, tumor recurrence was found in 80.8% of patients in illuminated regions or at other sites (new primary tumors). The overall survival median was 31 months.

**Figure 10:**
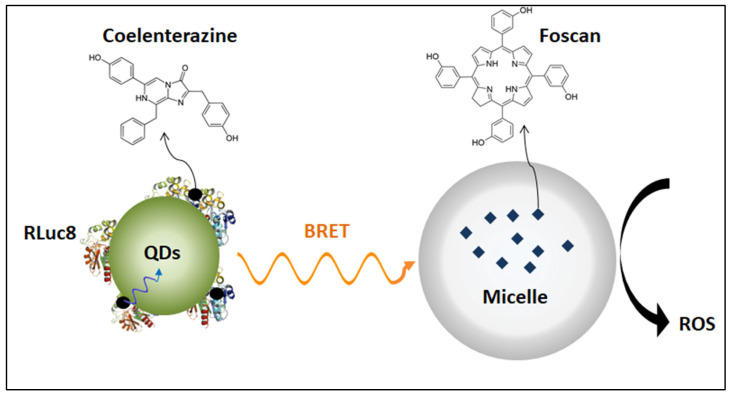
Self-illuminating quantum dots (QDs) interacting with photosensitizer-loaded micelle through BRET. Adapted from [66].

Although there are many clinical trials for Foscan (non-liposomal formulation) in cancer treatment, barely any clinical trials were conducted for Foslip^®^ (liposomal formulation). Only one clinical trial was conducted using Foslip^®^ in wound healing [65]; it was completed in 2018, but the results are not yet published. Foslip^®^ and its PEGylated formulation Fospeg^®^ are currently being researched extensively in-vivo for cancer treatment; please refer to Table 3 for more details. An interesting study was conducted by Hsu et al. [66] that uses Foscan^®^-loaded micelles in combination with self-illuminating quantum dots as an internal light source. The study utilized Renilla luciferase-immobilized quantum dots (QD-RLuc8) that self-illuminated at λ = 655 nm after being exposed to coelenterazine. Coelenterazine is the substrate of Renilla luciferase 8 (RLuc8), and when it is conjugated to carboxylate-containing quantum dots (QDs), energy is transferred from RLuc8 to QDs through bioluminescence resonance energy transfer (BRET), as shown in Figure 10 [66]. Hence, QDs will emit photons that can be used to activate Forscan^®^ in nearby micelles. The anti-tumor activity was assessed in-vivo using tumor-bearing mice (A549 lung cancer cells) using different treatment groups: Foscan^®^-loaded micelles + coelenterazine (m-F/coelenterazine), QD-RLuc8 + Foscan^®^-loaded micelles (QD-RLuc8/m-F), and the combination of QD-RLuc8, Foscan^®^-loaded micelles, coelenterazine (QD-RLuc8/m-F/ coelenterazine). Results showed a significant delay in tumor growth 20 days after treatment with the QD-RLuc8/m-F/coelenterazine formulation compared to other treatment groups. The technique of using a bioluminescent conjugate as an internal source of light inside the body can activate photosensitizers that are not accessible to external light when present in deep tumors.

## 4. Conclusions and Future Perspective

Light is relatively safe and has been used for many years in medical treatment, including photothermal therapy, photodynamic therapy, physiotherapy, and dentistry. In drug delivery systems, the ability to focus light on a specific body location allows a precise remote activation of light-sensitive nanocarriers in a spatiotemporal manner.

In this review, several mechanisms of drug release by light in photoresponsive nanocarriers were presented to qualitatively compare their mechanism in the spatiotemporal release of their payloads. Light can trigger drug release, with high control and precision, over a wide range of wavelengths from UV to NIR. In general, light-triggering mechanisms can be classified into three categories based on their functionality. First, a hydrophobic to hydrophilic change of a nanocarrier constituent(s) will make it more soluble and thus can disintegrate the intact structure of a nanocarrier (including micelles, liposomes or nanogels). For example, this transformation can be achieved by the Wolff rearrangement of DNQ molecules or the photo-isomerization of hydrophobic spiropyran (SP) to hydrophilic merocyanine (MC).

Second, introducing local defects within a nanocarrier can make the surface of the nanocarrier more porous or completely disrupt its intact structure. The *trans-cis* transformation of azobenzene or photo-cleavage of the nanocarrier lipids/polymers chains can achieve those local defects through *o*-nitrobenzyl or coumarin. Surface defects often increase the permeability of a therapeutic.

Third, metallic nanoparticles such as carefully constructed gold can induce heat upon irradiation with light which leads to temperature increases that release the payload of a nanocarrier. Thermal excursions can change hydrogen bonding interactions and create hydrophobic interactions, which change nanoparticle shape or increase permeability.

Despite the promising results in many in-vitro studies of photo-induced drug release from nanocarriers, many challenges limit light usage as a triggering mechanism. For example, UV light is associated with a high risk of tissue damage due to its high energy at short wavelengths. NIR irradiation is more preferable in drug delivery technologies, as it can penetrate deeper into tissues. However, the low energy of the NIR wavelengths may not be sufficient to induce a photochemical effect. To make NIR light more powerful in drug delivery, incorporating upconverting nanoparticles (UCNPs) into nanocarriers converts low-energy NIR irradiation to high-energy UV irradiation that cleaves specific molecules without directly irradiating with UV light. For deep tumors that cannot be reached by light at safe wavelengths, an internal source of light is necessary to induce photoreaction. One of the possible solutions is to insert a small optical fiber close to the tumor site. And another creative alternative is to use self-illuminating particles such as bioluminescent enzymes that can be used along with photosensitizer-loaded nanocarriers in photodynamic therapy.

## Figures and Tables

**Table 1:** Advantages and challenges of triggering stimuli used in drug delivery.

Stimulus	Advantage	Challenge	Reference
pH	∎ Distinct pH in tumor (pH = 6.5–7.2) compared to blood (pH = 7.35–7.45) and other organs. ∎ Intrinsically safe.	∎ Slow kinetics for drug release. ∎ Endogenous triggers are difficult to control (i.e., pH variation in tumor from one patient to another). ∎ A narrow range of pH variation poses a stability issue for nanocarriers.	[9,10,11]
Ultrasound (US)	∎ Non-ionizing safe radiation with deep penetration into tissue. ∎ Enhanced cell permeability and drug diffusion due to sonoporation. ∎ Potential for image-guided treatment. ∎ Easy to control.	∎ US-responsive medium (gas/PFC) is required. ∎ Possible tissue damage by heat for high-intensity US. ∎ Possible tissue damage by irreversible pore formation in cell membranes.	[11,12,13]
Light	∎ Potential to be highly focused more than other stimuli (e.g., ultrasound and magnetic field). ∎ A wide range of operating wavelengths: UV, visible, and NIR.	∎ Low penetration into tissue by UV and visible light. ∎ NIR penetrates tissue deeper but with lower energy which may not induce phototriggering from nanocarriers. ∎ The energy released from nanocarriers that require high light dosage can thermally damage tissues.	[14,15]
Magnetic Field	∎ Safe with high precision. Tissues with accumulated magnetic nanoparticles will only be affected while nearby tissues remain transparent to the magnetic field. ∎ Potential for magnetically-guided drug targeting through contrast agents. ∎ Drug release from thermo-sensitive nanocarriers by hyperthermia via an alternating magnetic field.	∎ Potential toxicity from metals (e.g., ROS from iron oxide decomposition). ∎ Expensive and complex equipment set-up that requires high expertise. ∎ A high external magnetic field is required to induce magnetism, especially in deep tissues. ∎ Difficulty to focus alternating magnetic field.	[11,16,17,18]
Hyperthermia	∎ Enhanced tumor permeability. ∎ Reduced hypoxic conditions and increased blood flow and drug delivery. ∎ Synergistic effect. Apoptosis of cancer cells is sensitive to hyperthermia. ∎ Effective in transdermal administration.	∎ Difficult to spatially control hyperthermia (~40–42 °C) at the tumor. ∎ Risk of superficial tissue damage by heat.	[19,20,21]

**Table 2:** Photo-triggered drug release in-vitro studies.

Mechanism	Nanocarrier	Loaded/Conjugated Drug	Active Moiety	Light Wavelength	Reference
photo-isomerization	Liposome (AzoC_10_N^+^/Schol)	Sulforhodamine B	Monoacylated azobenzene amphiphile	350 nm	[45]
	Micelle (SP-*hb*-PG)	Pyrene	Spiropyran	254 nm	[46]
	Micelle (SP-PMPC)	Doxorubicin	Spiropyran	365 nm	[25]
	Micelle (PEG-DASA)	Nile Red Paclitaxel	Donor–acceptor Stenhouse adducts (DASA)	Visible light	[47]
Photo-cleavage	Liposome (PC/Chol)	Co-loaded with doxorubicin hydrochloride + ZnPcRLA	ZnPcRLA	685 nm	[48]
	Liposome (DOPC/EYPC/Chol)	Basic orange 14 Doxorubicin	Cationic amphiphilic phthalocyanine	665 nm	[49]
	Liposome (NVOC-DOPE)	Calcein	NVOC-DOPE	λ > 300 nm (UV)	[50]
	Liposome (Egg PC)	5(6)-carboxyfluorescein	Hydrophobically modified poly(vinyl alcohol)-epoxypropoxy coumarin	254 nm	[51]
	Micelle (PCL-ONB-SS-PMAA)	Doxorubicin	*o*-nitrobenzyl ester	365 nm	[52]
Surface plasmon resonance absorption	Gold nanoparticles	Doxorubicin	Gold nanoparticles	660 nm	[53]
	Liposome (DPPC/DSPE-PEG2000)	Doxorubicin	Gold nanoparticles	660 nm	[54]
	Liposome (DPPC/DSPC/DSPE-PEG2000)	Calcein	Gold nanoparticles	656 nm850 nm	[55]

**Table 3:** Photosensitizer-loaded nanomedicine in-vivo studies.

Photosensitizer	Nanocarrier	Tumor Model	Reference
Foslip^®^ (m-THPC)	Liposome (DPPC, DPPG)	HT29 (human-derived colon adenocarcinoma cell line)	[67]
		143B (human-derived osteosarcoma cell line)	[68]
		CAL-33 (human-derived tongue squamous carcinoma cell line)	[69]
Fospeg^®^ (m-THPC)	Liposome (DPPC, DPPG, PEG-DSPE)	HT29 (human-derived colon adenocarcinoma cells)	[67]
		143B (human-derived osteosarcoma cell line) & K7M2L2 (mouse-derived osteosarcoma cell line)	[68]
		MC28 (methylcholanthrene-induced fibrosarcoma cell line)	[70]
Foscan (m-THPC)	Lipidots nanoemulsion (Lecithin-PEG)	CAL-33 (human-derived tongue squamous carcinoma cell line)	[69]
	Polymeric micelles (P(CL-TMC-Bz)-PEG) and (Bz-PCL-PEG)	A431 (human-derived squamous cell line)	[71]
Visudyne^®^ (verteporfin)	Liposomes (DMPC, EPG)	Meth-A sarcoma	[62]
		A431 (human-derived squamous cell line)	[72]

## Data Availability

No new data were created or analyzed in this study. Data sharing is not applicable to this article.
